# Long-Term, High-Dose Intravenous Immunoglobulin Therapy in a Patient with Banker-Type Juvenile Dermatomyositis

**DOI:** 10.1007/s12013-014-9833-7

**Published:** 2014-02-06

**Authors:** George Imataka, Osamu Arisaka

**Affiliations:** Department of Pediatrics, Dokkyo Medical University School of Medicine, 880 Kitakobayashi, Mibu, Shimotsuga, Tochigi 321-0293 Japan

**Keywords:** Juvenile dermatomyositis, Steroid, Immunoglobulin, Banker type

## Abstract

Juvenile dermatomyositis (JDM) is a multisystem disease characterized by non-purulent inflammation in the striated muscle and skin. Banker-type JDM is difficult to treat and control, especially when it is a form that is resistant to steroid treatment. Here, we report a 2-year-old girl with Banker-type JDM resistant to steroid treatment. The patient received intravenous immunoglobulin (IVIg) at 400 mg/kg/5 days a week every 6 weeks. Motor function improved and the patient was able to walk after six cycles. IVIg was administered every four weeks for six cycles thereafter, and the patient was able to walk more quickly with an improvement in quality of life. No apparent adverse effects were observed during IVIg treatment.

## Introduction

Juvenile dermatomyositis (JDM) is an idiopathic, multisystem, collagen disease characterized by non-purulent inflammation in the striated muscle and skin. Clinical pathology differs from that of the adult type; there is a lower frequency of malignant tumors and many cases respond well to steroids. Clinically, JDM can be classified into three subtypes: Banker, Brunsting, and fulminant [1]. There is no established therapy for steroid-resistant Banker-type JDM, and symptoms are very difficult to control [1]. In the following case report, long-term, high-dose therapy, with intravenous immunoglobulin (IVIg), was performed in a 2-year-old girl with steroid-resistant, Banker-type JDM. This is the first reported case of IVIg treatment in a child as young as 2 years old.

## Case report

A 2-year-old girl was brought to the hospital because of a waddling gait with intermittent claudication. In addition, she was not able to stand on one leg. Manual muscle testing (MMT) of the deltoid and quadriceps femoris resulted in a grade of three, showing muscle weakness predominantly in the proximal muscles. Also, Gower’s sign was positive. Muscle tonus did not restrict the range of motion of joints, and muscle mass was normal, but the gastrocnemius was hard and painful to the touch. Erythema and heliotrope-like exanthema were noted in the forehead. Upon neurophysiological examination, motor and sensory nerves were deemed normal. Muscle CT showed partial fatty degeneration in the gastrocnemius. Blood test results were as follows: creatine phosphokinase (CPK) = 14,420 IU/l; myoglobin = 777 ng/ml; aspartate aminotransferase (AST) = 211 IU/l; antinuclear antibodies (ANA) = 5,120 times; and negative for the Jo-1 antibody. Upon biceps brachii muscle biopsy, necrosis, regeneration and irregular sized myofibers, and angitis accompanied by perivascular lymphocyte infiltration were noted. JDM was diagnosed based on various tests and the clinical course.

After the initial oral prednisolone (PSL) dose at 2 mg/kg/day, CPK decreased to about 1,000 IU/l after 1.5 months; however, glaucoma developed after several months. Dual-energy X-ray absorptiometry index was 0.3. The PSL dose was gradually reduced, and CPK increased to more than 8,000 when the dose was reduced to 1 mg/kg/day, indicating recurrence of condition. Clinically, the patient became unable to turn over in bed or move her legs against gravity. Muscle MRI showed fatty degeneration and atrophic changes predominantly in the gastrocnemius.

Administration of IVIg at 400 mg/kg/5 days every 6 weeks was initiated, and CPK levels stabilized between 4,000 and 5,000 IU/l. Although CPK did not decrease, motor function improved and the patient became able to walk after six cycles. IVIg was administered every four weeks for six cycles thereafter, and the patient was able to walk more quickly with an improvement in her quality of life. No apparent adverse effects were noted during IVIg treatment.

## Discussion

In the present case, standard steroid treatment initially exhibited an effect but the disease recurred during steroid tapering therapy. Thus, a 12-cycle long-term, high-dose IVIg was performed every 6 weeks for the first six cycles and every 4 weeks for the subsequent six cycles, focusing on immunological abnormalities in juvenile dermatomyositis. In our case, IVIg therapy resulted in clinical improvement in the patient’s muscle weakness. Subsequently, quality of life improved such that the patient was able to walk unassisted. No apparent adverse effects were noted during the course of IVIg therapy. IVIg therapy has recently been reported to be effective in the treatment of steroid-resistant JDM [1, 2]. In a retrospective analysis by Lam et al. [3] regarding IVIg therapy in patients with poor response to first-line corticosteroid therapy, it was found that IVIG may be effective in controlling JDM symptoms. However, no consensus has yet been reached on IVIg dosing intervals and doses. Interestingly, the patient’s daily physical activity levels increased with every 4 weeks of IVIg therapy. To date, long-term IVIg therapy for steroid-resistant JDM in a patient as young as two years of age has never been reported. Although the results of hematology in this case indicated that complete remission could not be realized, the results did suggest that IVIg therapy is useful for controlling steroid-resistant fulminant JDM in young children.
